# Development and psychometric evaluation of postgraduate nursing student academic satisfaction scale

**DOI:** 10.1002/nop2.727

**Published:** 2020-12-03

**Authors:** Pardis Rahmatpour, Hamid Peyrovi, Hamid Sharif Nia

**Affiliations:** ^1^ Department of nursing School of Nursing and Midwifery Iran University of Medical Sciences Tehran Iran; ^2^ Nursing Care Research Center/School of Nursing and Midwifery Iran University of Medical Sciences Tehran Iran; ^3^ School of Nursing and Midwifery, Amol Mazandaran University of Medical Sciences Sari Iran

**Keywords:** academic satisfaction, nurses, nursing, nursing student, postgraduate, psychometric, validation

## Abstract

**Aim:**

To develop and evaluate the psychometric properties of postgraduate nursing student academic satisfaction scale (PNSASS).

**Design:**

The mixed‐method study was carried out in two phases; (a) item generation by hybrid concept analysis and (b) item reduction by psychometric evaluation of the developed scale.

**Methods:**

The initial item pool (*N* = 209) was prepared based on concept analysis results and similar instruments. A total of 402 postgraduate nursing students willingly participated in online data gathering from August 2019 to May 2020. The validity (face, content and construct) and reliability (stability and internal consistency) of the scale were assessed.

**Results:**

Exploratory factor analysis identified that the scale had four factors which explained 64.80% of the total extracted variance. The results of confirmatory factor analysis showed a good model fit. The reliability of scale was strong to excellent. The results showed that the PNSASS has suitable validity and reliability properties, which can be used to measure the academic satisfaction of postgraduate nursing students.

## 1 INTRODUCTION

One of the most important indicators of accreditation of educational institutions is student satisfaction. It is obvious that students are the main stakeholders of universities, and the evaluation of their experiences is the most important component of the accreditation process and is essential for the positive image of universities (Onditi & Wechuli, 2017). In many universities around the world, annual student survey measures the level of satisfaction with the two main goals of helping the universities identify their weaknesses, as well as informing new students who are planning to enrol at university in the future (Dattey et al., 2019; Salmi & Saroyan, 2007).

In addition to the importance of satisfaction for the universities, it is also important for the students. Academic satisfaction is one of the concepts that is associated with many positive educational outcomes in students such as academic achievement, academic motivation, self‐efficacy and students' confidence (Haghdoost et al., 2015; Jamshidi et al., 2017; Noughani et al., 2015). Literature has pointed out that lower levels of student satisfaction lead to academic burnout, academic failure, anxiety or depression among students (Atalayin et al., 2015; Hakim, 2014).

Nursing is one of the most important academic programmes in the field of health, which graduates in this field should be competent, thoughtful and creative nurses with critical thinking and problem‐solving skills. Although nursing students’ learning is strongly associated with their satisfaction with courses, the literature showed that nursing students, who satisfied with their course, acquire new knowledge, try to improve their clinical skills, build their professional profile and had a positive feeling about the future job (Alves & Raposo, 2007; Espeland & Indrehus, 2003; Hakim, 2014).

### 1.1 Background

In the academic context, student satisfaction refers to the “favorability of a student's subjective evaluation of the various outcomes and experiences associated with education” (Oliver & DeSARBO, 1989). Student academic satisfaction is a multifaceted concept (Gruber et al., 2010); several studies have been developing or testing student satisfaction models (Eom et al., 2006; Lent et al., 2007; Letcher & Neves, 2010) which could be concluded that all of them had considered four main components of the curriculum, teaching, social interaction and learning environment (Chen & Lo, 2015).

Despite the importance of the student satisfaction concept among all of the students, however, few studies have measured the level of satisfaction of postgraduate nursing students. In Iran, like elsewhere the world, a significant number of students are studying for PhD or master's degree and many undergraduate students plan to continue their education into a postgraduate degree in nursing (Hajihosseini et al., 2018; Wangensteen et al., 2018). Postgraduate nursing education curriculum in Iran is the same in all nursing schools and established by the Ministry of Health (Farsi et al., 2010). Also, the master's and PhD degree called postgraduate, and participation in this course requires a national entrance examination. Both the master and PhD course of nursing is fulltime and have thesis modules that are usually presented in the third and fourth semesters, respectively. Postgraduate nursing programmes have both theoretical and practical credits. In practical hours, postgraduate students experienced both the roles of learners and instructors or supervisors for undergraduate students in clinical settings (Moonaghi et al., 2017; Sajadi et al., 2017).

Due to the difference between postgraduate and the bachelor courses, the results of previous studies that focused more on undergraduate students (Dennison & El‐Masri, 2012; Smith et al., 2018) are not generalizable to postgraduate students. In addition, it should be noted that postgraduate students have higher levels of academic competence, are more mature than undergraduates and have a more advanced perspective about education (Muijs & Bokhove, 2017). They are faced with new challenges and different experiences such as doing the thesis/dissertation or research project dissertation (Muijs & Bokhove, 2017) and supervisor‐student interaction (Ahmadi et al., 2020; Mainhard et al., 2009). Unlike most undergraduate students who have to work in a clinical setting after graduation, postgraduate students have more job opportunities in the areas of research, education, policymaking, management and leadership (Haghdoost et al., 2015; Rautiainen & Vallimies‐Patomäki, 2016; Wilkinson et al., 2018).

Therefore, the different expectations and views of postgraduate students clarify the importance of explaining and measuring the concept of academic satisfaction among them. There are some student satisfaction scales with different dimensions that have been developed among students (Almeida et al., 2015; Asadizaker et al., 2015; Baptista et al., 2014; Chan et al., 2015; Chen et al., 2012; Dennison & El‐Masri, 2012; Franklin et al., 2014; Hirsch et al., 2016; Levett‐Jones et al., 2011), and however, they are not suitable for postgraduate nursing students. Regardless of the quality of development and psychometric properties of these scales (Rahmatpour et al., 2019), differences in the target population (undergraduate and associate degrees) were the reasons for not using these scales in the current study population.

Considering the importance of student satisfaction as an indicator for the quality of University educational performance and the need to pay more attention to postgraduate students’ academic expectations, this study is conducted to clarify the concept of academic satisfaction in postgraduate nursing students and then develop a valid and reliable scale to measure this concept accurately. The outcome of assessing student academic satisfaction will have benefits for both students and universities. It helps make the student's voice heard as the main stakeholders of the university. Also, these results could be useful for university managers to identify the strengths and weaknesses of their performance and appropriately allocate resources.

## 2 DESIGN

**FIGURE 1:**
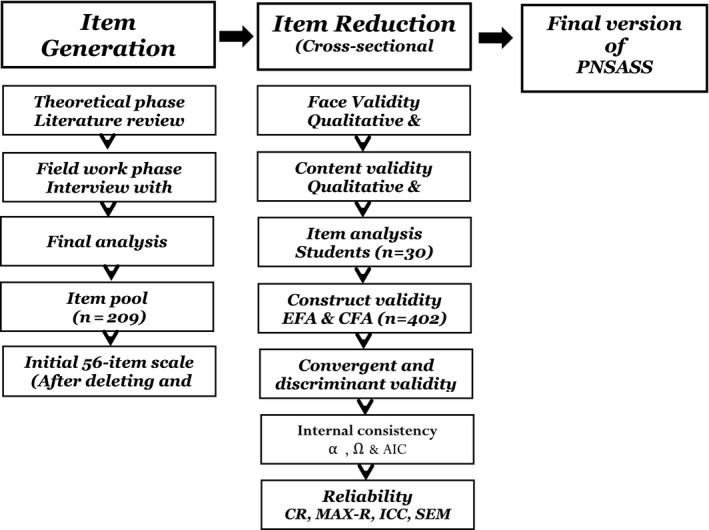
Flow chart of two phases of study

The sequential‐exploratory mixed‐method study was conducted from August 2019–May 2020 among Iranian postgraduate nursing students. It consisted of two phases: item generation by a hybrid concept analysis and item reduction by a cross‐sectional design for evaluating the psychometric properties of the developed scale (Figure 1).

## 3 METHODS

### 3.1 Item generation

A hybrid concept analysis of postgraduate nursing student academic satisfaction was used to elaborate postgraduate nursing students’ viewpoints along with a literature review for a better and more reliable understanding of the concept (Schwartz‐Barcott & Kim, 2000). The hybrid model includes three phases: theoretical, fieldwork and final analysis.

In the *theoretical phase*, electronic databases such as PubMed, Scopus, ISI web of science, ERIC and Persian databases were searched out with the keywords “academic satisfaction,” “student satisfaction,” “higher education,” “postgraduate” and “nursing student” in publications until 2019. Peer review published articles in English and Persian language were included. The exclusion criteria were editorial and commentarial materials and articles that full texts were not available. In the initial search, 753 studies (709 English and 44 Persian languages) were obtained, after excluding duplicated and irrelevant studies, 40 studies (33 English and seven Persian languages) were included and the conventional content analysis method according to steps proposed by Graneheim and Lundman (2004) was used to extract initial codes. Similar initial codes (*N* = 217) were classified as categories and subcategories of attributes, antecedents and consequences of the concept.

In the *fieldwork phase*, to reach a deep understanding of the concept, individual, face‐to‐face, in‐depth and semi‐structured interviews (30–90 min) were conducted from September–December 2019. Ten postgraduate students were recruited as participants by purposeful sampling (six PhD and four master students, 70% were female, mean age 32.10: *SD* = 6.15 years old). According to (Schwartz‐Barcott & Kim, 2000), three to six individuals are appropriate for the hybrid model. The interview questions were in line with the attributes, antecedents and consequences categories that formed in the theoretical phase. All the interviews were recorded. After transcribing recorded data, the texts of interviews were analysed with a directed content analysis method using MAXQDA software Ver.10. The directed approach to content analysis was used to validate or extend conceptually a theoretical framework (Hsieh & Shannon, 2005).

In the *final analytical phase*, the results of the fieldwork phase were compared with the data gathered from the literature review in the theoretical phase. According to (Schwartz‐Barcott & Kim, 2000) in some cases, results of fieldwork confirmed the categories formed in the theoretical phase or create new ones that ultimately lead to the refinement and development of the concept. In this study, all attributes, antecedents and consequences of the concept in the theoretical phase were confirmed and also three subcategories and one category were added in antecedents of concept. To ensure the trustworthiness of qualitative data used, the Guba criteria, including credibility, confirmability, dependability and transferability were considered (Guba, 1981).

Finally, based on the categories, subcategories of the concept analysis, and reviewing the existing related instruments, the main dimensions of the scale were determined. Based on the extracted codes, appropriate phrases were developed in each dimension and an item pool (*N* = 209) was developed. During frequent meetings of the research team, writing and grammar, overlap and similarity of items were checked, and some items were merged or deleted. Thus, the number of items was reduced from 209–132 items and then to 56 items. Finally, the 56‐item Postgraduate Nursing Student Academic Satisfaction Scale (PNSASS) was prepared to be evaluated in terms of psychometric properties.

### 3.2 Item reduction

The psychometric properties of the PNSASS with a seven‐point Likert response scale (completely agree to completely disagree) were assessed in terms of face, content and construct validity, and reliability.

#### 3.2.1 Face validity

Qualitative and quantitative face validity was used for the PNSASS. Ten postgraduate nursing students (Master = 5, PhD = 5) volunteered to conduct face validity testing on the 56‐item scale. For qualitative face validity, items were examined in terms of difficulty, relevancy and ambiguity. According to the participants’ viewpoints, necessary corrections were made for some items.

In quantitative face validity, the same ten students were asked to select one of five following responses for each item: “it is completely important, it is important, it is almost important, it is a little important, it is not important at all.” The scores were between 1–5, where the score of 1 reflected the lowest and the score of 5 the highest importance. The impact score was calculated for each item and score >1.5 were considered acceptable (impact score = frequency (%) × importance). All items in this section received an impact score above 1.5, which was acceptable.

#### 3.2.2 Content validity

In qualitative content validity, experts in the field of nursing and medical education (*N* = 14) were asked to assess grammar and wording of items, item allocation and scaling. According to their feedback, the items were modified. Then, content validity ratio (CVR) was examined to evaluate items’ necessity (unnecessary = 1, somewhat necessary = 2, necessary = 3). Since the number of experts was 14, the minimum acceptable CVR score based on Lawshe was considered equal to 0.51 (Lawshe, 1975). At this stage, five items were removed (CVR < 0.51) and two items were divided into two parts, which finally reduced the number of items from 56–53. Items relevancy (dichotomous response: relevant = 4, irrelevant = 1) of the 53‐items scale was evaluated by 11 experts. For the elimination of chance effect, modified Kappa was calculated for each item (good = 0.60–0.74 and the excellent value of Kappa > 0.74; Ebadi et al., 2020). All items had an acceptable kappa value.

#### 3.2.3 Item analysis

Before examining the construct validity, an item analysis was conducted with 30 postgraduate nursing students (20 PhD and 10 master students, 62% were female, mean age 30.96 [*SD* = 5.9] years old) as participants to identify the possible problems of items and to compute the inter‐item correlation. Items with the correlation coefficient between items lower than 0.3 were removed. Additionally, if Cronbach's alpha was increased with the removal of an item, it showed that the item should be deleted. At this stage, the total and standardized Cronbach's alpha was 0.966 and 0.967 respectively, which is acceptable (Ebadi et al., 2020). Five items had a correlation coefficient of 0.32 and lower and were removed, and the number of items was reduced to 48 items.

#### 3.2.4 Construct validity

The construct validity of the PNSASS with 48 items was assessed through maximum‐likelihood exploratory factor analysis (MLEFA) method and with Promax rotation. Sample adequacy was estimated through the Kaiser–Meyer–Olkin (KMO) and Bartlett's tests. KMO values of higher than 0.9 were interpreted as excellent (Pahlevan Sharif & Sharif Nia, 2020). The sample size for factor analysis was estimated using the rule of thumb that considers 200 participants as an adequate sample size (MacCallum et al., 1999). In this study, two independent samples were collected, 200 for exploratory factor analysis (EFA) and a second 202 sample to evaluate confirmatory factor analysis (CFA). In total, 402 postgraduate nursing students were recruited into the study. The demographic characteristics and educational information of participants are presented in Table 1.

**TABLE 1:** Demographic characteristics and educational‐related information of participants (*N* = 402)

Variables	*N* (%)
Gender	
Female	255 (63.4)
Male	147 (36.6)
Marital status	
Single	195 (48.5)
Married	207 (51.5)
Education degree	
PhD	172 (42.8)
Master	230 (57.2)
Accommodation	
Home	219 (54.5)
Private dormitory	51 (12.7)
University dormitory	132 (32.8)
Employment status	
Employed	217 (54.0)
Unemployed	185 (46)
Age (mean ± *SD*) years	32.05 ± 6.0
GPA (mean ± *SD*) of 20	17.60 ± 1.3

The online data gathering was performed for this section. The online questionnaire was created via Google Form and its URL link was sent by email or social networking applications such as Telegram channel or WhatsApp group of nursing postgraduate students. Data were extracted in the Excel file from Google Form and prepared for analysis. The presence of an item in a latent factor was determined based on a factor loading of almost 0.3, which was estimated using the following formula: CV = 5.152 ÷ √ (*n* – 2), where CV was the number of extractable factors and “*N*” was the sample size (Norman & Streiner, 2008). The number of latent factors was estimated using Horn's parallel analysis (Çokluk & Koçak, 2016). Next, items with communalities <0.2 were excluded from EFA (Hahs‐Vaughn, 2016). For assessment of the structural factors, CFA was conducted using the maximum‐likelihood method and the most common goodness‐of‐fit indices. The model fitness was assessed according to root mean square of error of approximation (RMSEA), parsimonious normed fit index (PNFI), parsimonious comparative fit index (PCFI), Tucker–Lewis index (TLI), comparative fit index (CFI), incremental fit index (IFI) and CMIN/DF.

#### 3.2.5 Convergent and discriminant validity

Average variance extracted (AVE) and maximum shared squared variance (MSV) and composite reliability (CR) were estimated to assess the convergent and discriminant validity of the extracted factors. To establish convergent validity: (a) AVE should be >0.5 and (b) CR should be greater than AVE. To meet the discriminant validity, MSV for each construct should be less than AVE (Ahadzadeh et al., 2015).

#### 3.2.6 Reliability

Internal consistency was assessed by Cronbach's alpha (*α*), McDonald's omega (Ω) and the average inter‐item correlation (AIC). Coefficient's *α* and Ω values >0.7 (Mayers, 2013) and AIC 0.2–0.4 was considered as an acceptable internal consistency (Mohammadbeigi et al., 2015). CR and maximum reliability (Max H reliability) which replaces Cronbach's alpha coefficient in structural equation modelling was then evaluated, and value >0.7 were considered acceptable (Sharif Nia et al., 2019). The stability of the PNSASS was measured by intra‐class correlation coefficients (ICC; Javali & Gudaganavar, 2011). ICC was used with two weeks interval in 30 postgraduate nursing students by using a two‐way random effect. Moreover, standard error of measurement (*SEM*) that is measuring errors of scale score was calculated for the scale (*SEM* = *SD*_Pooled_ × √1 − ICC).

#### 3.2.7 Multivariate normality and outliers

Univariate distributions were examined for outliers, skewness and kurtosis. Multivariate distributions were evaluated for normality and multivariate outliers. Multivariate normality can be evaluated through the use of Mardia's coefficient of multivariate kurtosis. One indication of deviation from normal distribution was a Mardia's coefficient >8. Multivariate outliers were evaluated through the evaluation of a Mahalanobis distance. Items with a Mahalanobis distance of *p* < .001 were considered to be multivariate outliers (Esposito Vinzi et al., 2010). All of the statistical procedures were analysed by SPSS_26_AMOS_24_, the SPSS R‐Menu_2.0_ and JASP_0.13.0.1_.

#### 3.2.8 Ethical consideration

The protocol of this study was approved by the Iran University of Medical Sciences Research Ethics Committee (IR.IUMS.REC.1397.1311). In all steps of this study, participants were informed about the purpose of the study.

Before conducting the interview, oral informed consent was obtained from the participants. The participants were allowed to leave the study at any time. All participants were assured that recorded interviews would be kept private and results would be reported anonymously.

Also, in the online survey, all information required in a consent form was explained in the introduction part of the online questionnaire; the study aims, the number of items, time for completing the scale, the researcher's affiliation and email for queries and ethical code of study and also we informed participants that their participation was voluntary and that their responses would be published anonymously as group data. The online questionnaire items are not viewed by participants until they agree to participate and click on the “next button.”

## 4 RESULTS

### 4.1 Item generation

Based on the combination of results of theoretical and field‐work phases of the concept analysis, the concept of academic satisfaction in postgraduate nursing students had five attributes (multidimensional, resultant nature, unique, student‐based and positive feeling), seven antecedents (purposeful academic teaching, student characteristics, professors characteristics, positive academic relationship, high quality of educational services, perceived value, nursing vision) and three consequences (academic performance, university reputation, overall satisfaction). These categories were considered as scale domains with 209 items. Out of which 56 items were allocated for the PNSASS.

### 4.2 Item reduction

After performing face and content validity, the number of scale items decreased from 56–53. Following the item analysis, five items were removed, and the 48‐item scale entered the factor analysis step. In MLEFA, the KMO test value was 0.957 and Bartlett's test value was 6,260.613 (*p* < .001). Four factors were extracted (32 items) and named as “nursing curriculum” (14 items), “academic interactions” (12 items), “teaching and learning” (three items) and “educational facilities” (three items). These four factors explained 64.80% of the total variance of academic satisfaction among postgraduate nursing students (Table 2).

**TABLE 2:** The four factors of the PNSASS and their factor loadings (*N* = 200)

Factors	Items	Factor loading	*h* ^2^	*λ*	% Variance
Nursing curriculum	24. The curriculum content is changed proportionally to the needs of the student	0.986	0.721	6.68	20.90
	23. The curriculum content is commensurate with the expected competencies of nursing graduates	0.845	0.680		
	28. the curriculum helps to improve student required professional skills	0.811	0.675		
	21. During this course, student educational abilities are well used	0.787	0.653		
	22. During this course, student research abilities are well used	0.746	0.672		
	20. There is a dynamic atmosphere for learning and research in the faculty	0.663	0.602		
	43. Recreation and welfare facilities are provided for students	0.658	0.376		
	26. Assignments of this course are appropriate for the course hours and units	0.619	0.631		
	27. The evaluation criteria for student academic performance are well defined	0.596	0.618		
	25. The curriculum content is properly implemented	0.595	0.683		
	13. Professors provide student communication with specialists in other fields	0.580	0.545		
	6. During this course, the student has an opportunity to carry out extracurricular activities	0.554	0.413		
	17. Faculty academic planning and scheduling are well organized	0.473	0.491		
	7. Clinical training helps to improve my clinical skills (critical thinking, responsibility, and etc.)	0.457	0.339		
Academic interactions	35. There is a proper interaction between me and professors.	0.968	0.796	6.54	20.46
	37. There is a proper interaction between me and faculty staff	0.863	0.622		
	36. There is a proper interaction between me and the faculty administrators	0.850	0.703		
	34. Instructors comprehend my issues and concerns.	0.809	0.746		
	33. Student has a friendly relationship with classmates	0.671	0.379		
	10. Professors have a sense of commitment and responsibility to students	0.646	0.651		
	31. interactions in the faculty are professional and respectful	0.605	0.672		
	15. Student is free to choose and act on issues related to dissertation/thesis (choosing a topic or supervisor, etc.)	0.521	0.510		
	8. Professors are available during office hours	0.515	0.467		
	12. Professors are fair and unbiased in their treatment of individual students	0.432	0.547		
	32. There is a positive competitive atmosphere among students	0.399	0.482		
	44. I feel good taking this course	0.381	0.544		
Teaching and learning	3. The teaching method leads to effective learning in students	0.849	0.858	4.77	14.92
	2. Expert and experienced professors are training student	0.833	0.799		
	1. Classroom teaching has the required quality	0.766	0.759		
Educational facilities	41. Educational technologies (such as video projectors, whiteboards and, etc.) are desirable in the classroom	0.761	0.696	2.72	8.51
	42. The library possesses a sufficient number of up‐to‐date textbooks	0.725	0.583		
	38. The educational environment (classroom, clinical setting, study room for graduate students and, etc.) has a high quality	0.633	0.629		

Abbreviations: *h* ^2^, item communality, λ, eigenvalue.

**FIGURE 2:**
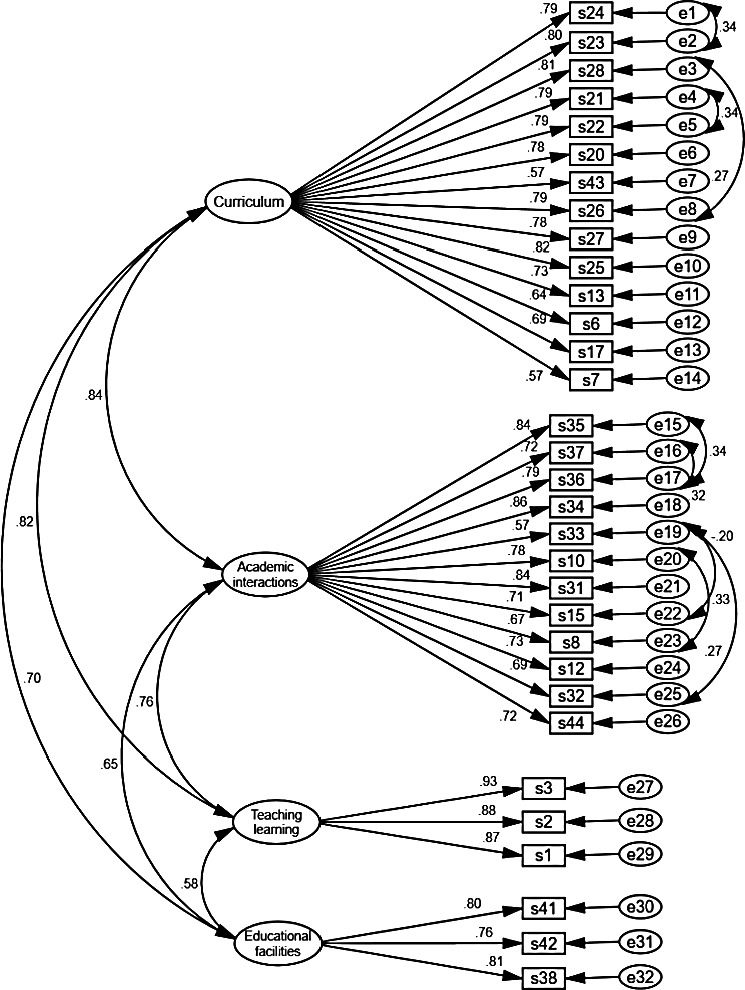
The PNSASS construct: modified model of first‐order confirmatory factor analysis (*N* = 202)

**FIGURE 3:**
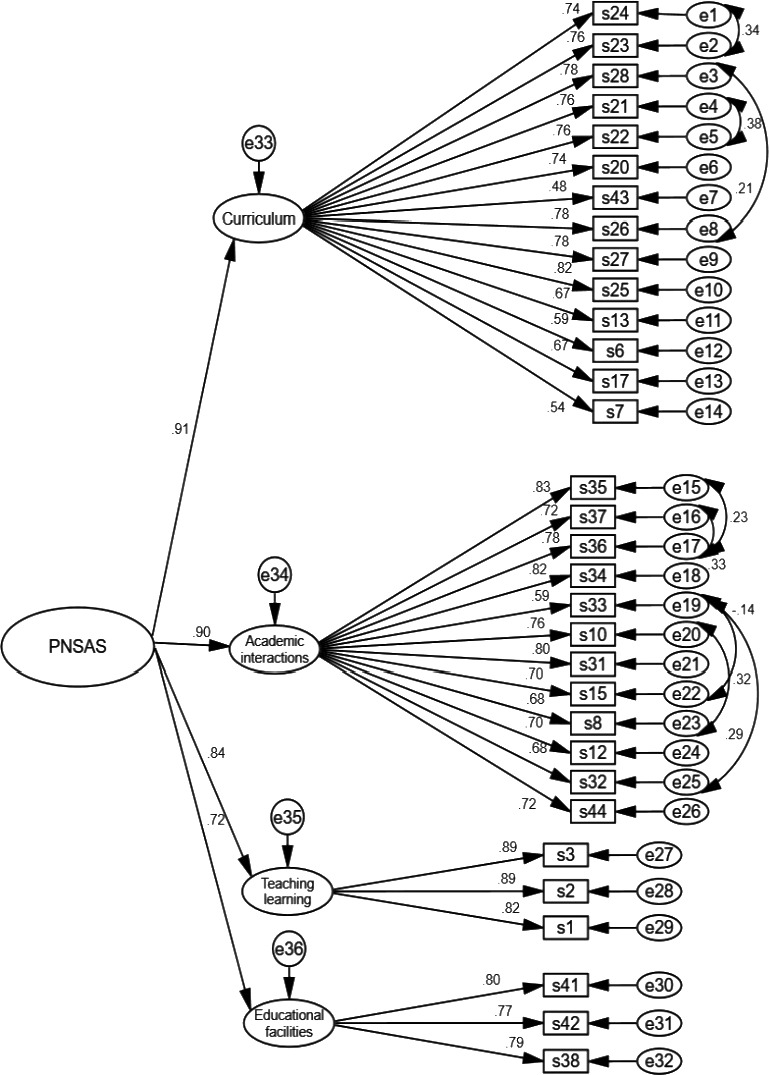
The PNSASS construct: modified model of second‐order confirmatory factor analysis (*N* = 202)

In the first‐order confirmatory factor analysis, after modification of the model (eight pairs of measurement errors between measured items of the *nursing curriculum* and *academic interactions* were allowed to freely covary), the chi‐square model fit index was calculated 949.18 (*p* < .001) and CMIN/DF = 2.109. Then, other model fit indices were calculated and these values confirmed the good fit of the final model (see Table 3). The co‐variances between factors were more than 0.50 that indicated a latent variable behind them (see Figure 2). So another assessment of the factors of the PNSASS and the correlation between them was performed. The second‐order CFA was conducted to confirm that a latent variable named postgraduate nursing students' academic satisfaction (PNSAS; see Table 3). Figure 3 shows the second‐order structural model and the CFA.

**TABLE 3:** Fit indices of the first‐ and second‐order confirmatory factor analysis of the PNSASS (*N* = 202)

Indices	*χ* ^2^	*df*	*p* Value	CMIN/DF	RMSEA	PNFI	PCFI	TLI	IFI	CFI
First‐order after structure modification	949.18	450	<.001	2.109	0.067	0.776	0.833	0.909	0.918	0.918
Second‐order after structure modification	962.91	453	<.001	2.126	0.068	0.779	0.837	0.908	0.917	0.916

Note

Fitness indexes: PNFI, PCFI (>0.5); TLI, IFI, CFI (>0.9), RMSEA (˂0.08), CMIN/DF (˂3 good, ˂5 acceptable).

Abbreviations: CFI, Comparative Fit Index; CMIN/DF, Minimum Discrepancy Function divided by Degrees of Freedom; *df*, degree of freedom; IFI, Incremental Fit Index; PCFI, Parsimonious Comparative Fit Index; PNFI, Parsimonious Normed Fit Index; RMSEA, Root Mean Square Error of Approximation; TLI, Tuker–Lewis Index.

The results of AVE, MSV and CR confirmed that in the first model of CFA the convergent validity was established but discriminant validity was not fulfilled (see Table 4). The MSV for factors one and two were not less than AVE. This confirmed that the extracted factors are not separate from each other and the running the second‐order CFA is required.

**TABLE 4:** The indices of the convergent, discriminant validity and internal consistency of PNSASS for the first‐order CFA (*N* = 202)

Factors	Indices						
	AVE	MSV	CR	MaxR (H)	Alpha (CI 95%)	AIC	Omega
Nursing curriculum	0.553	0.714	0.945	0.950	0.942 (0.931–0.952)	0.540	0.943
Academic interactions	0.559	0.714	0.938	0.946	0.937 (0.925–0.947)	0.555	0.938
Teaching and learning	0.803	0.669	0.924	0.929	0.915 (0.896–0.932)	0.753	0.917
Educational facilities	0.620	0.496	0.830	0.832	0.825 (0.786–0.859)	0.612	0.826

Abbreviations: AIC, Average Inter‐item Correlation; Alpha, Cronbach's alpha; AVE, Average Variance Extracted; CR, Composite Reliability; MaxR (H), maximum reliability; MSV, Maximum Shared Squared Variance; Omega, McDonald's omega coefficient.

Internal consistency of scale revealed that Cronbach's alpha, McDonald's omega and AIC of all factors were greater than 0.7 and 0.4, respectively. In addition, CR and maximum reliability (Max H) of factors showed that there was a strong coefficient (Table 4). The overall ICC for PNSASS was 0.923 (CI 95: 0.91–0.93) that indicated a strong stability of the scale over time. The value of *SEM* for the scale was ±7.82 which indicated the individuals’ scores on the same scale tend to be distributed 7.82 value around their “true” score.

## 5 DISCUSSION

The results of this study supported that the final PNSASS had the desired validity and reliability, included 32 items and four factors namely nursing curriculum, academic interactions, teaching and learning, and educational facilities which explained 64.80% of the total extracted variance. In this study, CFA was used and confirmed the PNSASS model fitness. A high correlation between the first‐order constructs displays that the latent variables do not completely act as an independent variable and their correlation reflects the presence of a more general construct at a secondary conceptual level. According to the findings of second‐order CFA, the correlation of the domains with the total scale and the low correlation of the domains with each other indicated that none of the domains was exactly the same.

The goal of factor extraction is to maximize explained variance (Polit & Yang, 2015) that in this study was 64.80%. The greatest values of the explained variance belonged to the curriculum factor (20.90%) and academic interactions factor (20.46%). Previous studies that developed student satisfaction scales regardless of the factor extraction method, reported smaller explained variance than the PNSASS. Undergraduate nursing student academic satisfaction scale (UNSASS) with 48 items explained 50.12% of the total variance (Dennison & El‐Masri, 2012) and the nursing student satisfaction scale (NSSS) with 27 items had 55.7% explained variance (Chen et al., 2012).

According to the results of Cronbach's alpha, AIC and McDonald's omega, the PNSASS demonstrated strong and excellent internal consistency. Also, the PNSASS possesses strong stability with the acceptable value of ICC, which is one of the advantages of this scale. In this study, *SEM* of the scale was calculated. Measurement error is an important and required domain of COSMIN (COnsensus‐based Standards for the selection of health Measurement Instruments; Terwee et al., 2012). The smaller value of *SEM* of the scale is very important. Indeed, *SEM* quantifies the accuracy of the score of any individual. Previous psychometric evaluation studies of the student satisfaction scale did not report this valuable index.

Based on the factor loading of items in the EFA, four factors extracted, namely “nursing curriculum,” “academic interactions,” “teaching and learning” and “educational facilities,” are discussed below.

The first extracted factor was labelled the *nursing curriculum*. It comprised 14 items reflecting curriculum contents (assignments, students’ skills, evaluation criteria, clinical programme, syllabus, etc.). In many related scales, this factor has been extracted as an important domain (Chen & Lo, 2015; Dennison & El‐Masri, 2012; Lai et al., 2015). According to Ali et al. (2016), among all dimensions a robust and flexible curriculum is most important in forming the perceptions of service quality that affect student satisfaction. Dennison and Maher believed that measurement of satisfaction from a curriculum promotes a more meaningful assessment of satisfaction with the entire nursing programme (Dennison & El‐Masri, 2012). Moreover, student evaluations of nursing curriculum content and syllabus had significant effects on improving students learning outcomes and their satisfaction (Chen & Lo, 2015).

The second extracted factor was *academic interactions* with 12 items, which addressed the student interactions with their classmates, professors, staff and administrators. The literature stated that the good and effective communication between the professors and the student contributes to the gradual development of self‐confidence and consequently in the achievement of students goals and also promotes greater effectiveness in education (Anagnostopoulou et al., 2015; Santos Neto et al., 2017). In addition, the interaction between the student and university personnel impact student learning and satisfaction (Chen & Lo, 2015; Elliott & Shin, 2002). Shahsavar et al. emphasized that respectful and unbiased behaviour of administrative and university staff and caring about students are important factors in increasing student satisfaction (Butt & ur Rehman, 2010; Shahsavar & Sudzina, 2017).

The third factor was *teaching and learning*, with three items reflecting the effectiveness of the academic teaching method and expertise of professors in both class and clinical teaching. Several student satisfaction scale have this factor (El Ansari & Moseley, 2011; Nurunnabi & Abdelhadi, 2019). According to Butt and ur Rehman (2010) study, from the viewpoint of higher education students, professors’ expertise is the most influential factor with a positive impact on students’ satisfaction. Also, teaching method quality has been mentioned many times in the literature as a significant factor that influences overall student satisfaction (Burgess et al., 2018; Lai et al., 2015).

The last factor was labelled *educational facilities* consisting of three items referred to the educational environment, library and educational technologies. Although these are basic students’ needs for education, likely influence the teaching‐learning process. For this reason, extensive research has been carried out studying the educational facilities factors which can affect student academic satisfaction such as library facilities, a classroom environment that is the most value‐added contribution for university services among the students (Butt & ur Rehman, 2010; Lai et al., 2015).

### 5.1 Limitations

The sample was recruited from Iranian postgraduate nursing students, so the generalizability of the findings is limited. Despite the advantages of using an online questionnaire, lack of physical interaction, the inability to verify an individual's status and the veracity of their responses were limitations of this online survey.

### 5.2 Implication

The students’ viewpoints are important for University managers, for accurate planning, improving the quality of courses, and adapting the course to students' needs. A better understanding of students' concerns in terms of academic satisfaction enhances student motivation, and academic performance influences university reputation, and overall satisfaction of students. Thus, the PNSASS is a useful scale for university managers, researchers and students.

Moreover, the PNSASS with the fewer number of items, significant variance explained, as well as its narrow scope than the existing scales made it better differentiating and accurate measuring student academic satisfaction of postgraduate nursing students.

## 6 CONCLUSION

The results of this study revealed that the PNSASS consists of 32 items with four domains that had acceptable validity and reliability. Government and institutions should pay special attention to raise quality education and student academic satisfaction. Based on current study findings, a well‐designed curriculum, establishing interactive communication and a healthy climate between students, professors, staff and administrators, using qualified and expert professors for clinical and class teaching, providing a favourable learning environment is essential for postgraduate nursing students' academic satisfaction.

## CONFLICT OF INTEREST

None.

## ETHICAL APPROVAL

The protocol of this study was approved by the Iran University of Medical Sciences Research Ethics Committee (IR.IUMS.REC.1397.1311).

## Data Availability

The data sets generated for this study are available on reasonable request to the corresponding author.
